# Editorial: Bacterial Surface Glycans as the Virulence Agent and the Target for Predators, Therapy, and the Immune System

**DOI:** 10.3389/fmicb.2020.624964

**Published:** 2020-12-16

**Authors:** Zuzanna Drulis-Kawa

**Affiliations:** Department of Pathogen Biology and Immunology, Institute of Genetics and Microbiology, University of Wroclaw, Wrocław, Poland

**Keywords:** bacterial glycans, virulence, phage receptor recognition, enzymes degrading bacterial glycans, phage resistance mechanism

The Research Topic entitled “Bacterial surface glycans as the virulence agent and the target for predators, therapy, and the immune system,” was dedicated to reports and reviews on novel discoveries about bacterial glycans biodiversity, and recognition by the immune system, as well as about phage interactions with glycan receptors, and phage-derived enzymes recognizing/degrading bacterial glycans.

Bacterial surface molecules both of protein and carbohydrate nature such as type IV pili (T4P), outer membrane proteins, flagella, or capsule (CPS), exopolysaccharides (EPS), lipopolysaccharide (LPS), and peptidoglycan (PG) are targeted by bacterial predators (phages) as receptors. The successful phage adherence is accompanied by the passage of diverse carbohydrate barriers. For that purpose bacterial viruses are equipped with various virion-associated and soluble enzymes, called polysaccharide depolymerases and lysins, that recognize, bind, and degrade glycan compounds (Latka et al., 2017).

Nowadays, more and more papers describe the phage-encoded enzymes able to degrade CPS, EPS, or LPS of the host bacteria. Those are found not only within podoviruses but also in phages representing *Ackermannviridae, Myoviridae*, and *Siphoviridae* families. The minireview of Knecht et al., describes the diversity and function of different tail spike proteins (TSP) of phages propagating on the most common pathogens. It is an interesting work combining the current knowledge of known phages targeting single, dual, and multiple CPS/LPS serotypes of the bacterial host. Therefore, the authors propose possible systems connecting TSPs with the baseplate in different phage genera to enlarge phage specificity to more than one type of targeted glycan.

The above idea has been further continued in detail in the model of *Klebsiella* phages (Latka et al.). This work is dedicated to *in silico* modeling of Receptor Binding Proteins (RBPs) architecture based on the experimental structural results done previously by Leiman group in *E. coli* phages. A detailed bioinformatic analysis shows that RBPs are structured with conservative and divergent domains. The N-terminal domain responsible for RBP assembly to the virion/baseplate may be complemented with a specific T4gp10-like domain providing the attachment site for the secondary RBP in a so-called branching system. The *in silico* modeling shows the possible commitment of the T4gp10-like domain in the RBPs architecture of multi-serotype targeting *Klebsiella* phages. Moreover, this idea has been lately confirmed by this group in the structural and functional study on *Klebsiella* phage capsule-degrading depolymerase KP32gp38.

The second type of phage-borne enzymes as potential antibacterials are lysins, peptidoglycan-degrading proteins. These enzymes are efficiently applied against Gram-positive bacteria whereas with some limitations against Gram-negatives because of the protective outer membrane layer blocking the access to targeted peptidoglycan. To overcome this obstacle lysins can be combined with membrane-disrupting agents. The work of Ciepluch et al. describes the pros and cons of dendronized silver nanoparticles utilization as bacterial membrane permeabilizers together with lysins against *P. aeruginosa*. It turned out that cationic nanoparticles were interacting with the O-chain of LPS with no direct access to the OM surface. Only the dendronized AgNPs PEGylation overcame the LPS barrier and efficiently enhanced the antibacterial effect of lysins.

In this Research Topic, we were looking for current findings referring to phage interactions with glycan receptors to see if the naturally occurring heterogenicity in surface polysaccharides impacts the phage predation. On the other hand, the phage introduction might select a phage-resistance in the targeted host leading to the modification of a specific phage receptor. In other words, the variation of the capsule, LPS, or other types of surface glycan, the main molecular patterns for the immune system recognition would entail the diminishing virulence of bacterial pathogens.

Indeed, the work of Cai et al., shows the correlation between the spontaneous mutations in the genes of three capsular polysaccharide synthesis-related glucosyltransferases (GT-1, GT-2, and WcaJ) and both the prevention of phage GH-K3 infection and reduced virulence of *K. pneumoniae* K7. The rough-type phage-resistant mutant induced mitogen-activated protein kinase (MAPK) signaling pathway in macrophages becoming significantly more prone to endocytosis compared to the wild-type strain. The authors also found that phage GH-K3 was able to bound to modified CPS residues of K7(ΔGT-1) and K7(ΔwcaJ) mutants but with lower efficiency.

There is another very interesting mechanism of phage-bacterial glycan interaction in *Campylobacter jejuni* (Sacher et al.). This study was focused on the role of specific phage-encoded FlaGrab protein responsible for the phage attachment to the motile *C. jejuni* flagella. It turned out that FlaGrab recognizes the glycosylated flagella and triggers the bacterial response-signal inhibiting the growth and downregulating energy metabolism. It serves for the prevention of phage propagation. Moreover, it was proposed that the variation in flagella glycosylation provides resistance to phage infection. This study is a good starting point to get insight into unknown mechanisms of generating flagellar glycan diversity also in the context of *C. jejuni* virulence.

Bacteria had developed a panel of strategies to avoid the immune system response and phage attack. Indeed, the outer membrane vesicles (OMVs) bearing LPS molecules in the outer leaflet are used by Gram-negative bacteria as a perfect decoy attracting and sequestrating phages present in bacterial surroundings (Caruana and Walper, 2020). The manuscript of Stephan et al., presents the *in vitro* analysis of O-antigen-specific phage P22 inactivation by *Salmonella* Typhimurium OMVs. It was shown that phages were effectively neutralized by the deposition on the surface of the vesicles. A part of phages actively ejected their DNA into the OMVs interior and it was more intense in the presence of membrane proteins in native OMVs.

Glycoconjugates are the main bacterial virulence factors being at the same time molecular patterns as well as phage targets for efficient adsorption. Moreover, a high structure specificity of bacterial glycans may serve for bacterial identification, diagnostics, and vaccine compounds. The minireview prepared by Campanero-Rhodes et al. provides the view of the current state-of-the-art and prospects for the implementation of microarray strategies for exploring bacterial surface glycans and their interactions with glycan-binding proteins. This sensitive and high-throughput system allows for fast identification and differentiation of glycan-protein interactions further used in bacterial diagnostics, the immune system effectors recognition, glycan-degrading enzymes detection as well as vaccine development. The different microarray platforms may incorporate glycan molecules, cell wall fragments, or entire bacterial cells as a template.

Summarizing the content of this Special Issue focused on bacterial glycans as the key element both for bacterial virulence as well as phage targeted receptors, we may conclude that co-evolutionary interactions between bacteria and its host, bacteria and its predators forced microorganisms to develop sophisticated strategies to avoid the immune response and viral propagation. The deepened knowledge on bacterial glycan variability and glycan-directed phage specificity will allow us to understand the evolutionary linkages and mutual bacteria-phage impact on biodiversity, as well as in the context of future application perspectives in medicine, industry, agriculture, or biotechnology.

## Author Contributions

ZD-K wrote the editorial note.

## Conflict of Interest

The author declares that the research was conducted in the absence of any commercial or financial relationships that could be construed as a potential conflict of interest.
